# Detection of a numeric value predictive of increased dose to left anterior descending coronary artery (LAD) in radiotherapy of breast cancer

**DOI:** 10.1186/s40064-016-2399-7

**Published:** 2016-06-23

**Authors:** R. Di Franco, V. Ravo, V. Nieddu, A. Crispo, S. Falivene, F. M. Giugliano, A. Argenone, V. Borzillo, F. Cammarota, P. Muto

**Affiliations:** UOC Radiation Oncology, Istituto Nazionale per lo studio e la cura dei tumori “Fondazione Giovanni Pascale” – IRCCS, Via Mariano Semmola, 80131 Naples, Italy; Division of Epidemiology, Istituto Nazionale per lo studio e la cura dei tumori “Fondazione Giovanni Pascale” – IRCCS, Via Mariano Semmola, 80131 Naples, Italy

**Keywords:** Radiotherapy, Breast cancer, Heart, Coronary arteries, Chest thorax

## Abstract

**Purpose:**

We have evaluated thoracic conformation of patients in order to derive a numeric value predictive of an increased dose to left anterior descending coronary artery (LAD), critical structure for the development of late radio induced cardiac morbidity.

**Methods:**

We have evaluated 91 patients (36–88 years) affected by breast cancer stage I–II (Tis–T1–2 N0–1), undergoing adjuvant radiotherapy with conventional fractionation. For each patient on CT images was measured the distance between the back face of the sternum (manubrium) and the anterior face of body of the corresponding vertebra (a), and the distance measured on the line at 45° between the vertebral body of the same vertebra and the back face of the rib corresponding (b). The a/b ratio showed values between 0.626 and 1.123. We used the median value (0.821) as cut-off to divide the patients in two groups. We calculated in both groups: Volume (Vol) heart, Vol LAD with an expansion of 0.6 mm; Dmean LAD (Gy); Dmax LAD (Gy); V10–V20–V30 (%) LAD and we correlated these values with parametric and non-parametric tests.

**Results:**

The Pearson test has showed a statistically significant correlation between Vol breast and V10, V20, V30 with borderline significance (p = 0.006; p = 0.02; p = 0.05). The data were confirmed by testing non-parametric Kendall (tau = 0.004; tau = 0.015; tau = 0.016) and Spearman (rho = 0.003; rho = 0.016; rho = 0.015). We conducted categorizing into quartiles of breast volume and evaluated the correlation with a/b. We have found a significative correlation (p = 0.01) between small Vol breast (≤660.23 cc) and a/b < 0.0821 and greater Vol breast (>660.23 cc) with a/b > 0.0821. From the evaluation of the distribution of V10 in the two groups taking account of the Dmean ≤5 or >5 significance was found with a/b; Chi square 0.009 (0.01). Values ≤5 were observed in women with a/b < 0.0821. Values >5 in women with a/b > 0.0821.

**Conclusions:**

The geometric conformity of chest thorax considering a/b and the value of 0.0821 can reveals an important parameter in the selection of patients suitable for radiation therapy on left breast in order to evaluate the risk of late cardiac events. This consideration during treatment planning can change the technique or the set-up allowing the development of a customized plan.

## Background

In early-stage breast cancer, the conserving therapy and whole breast radiation treatment are an the standard option for both local control of the tumor and for esthetic results (Early Breast Cancer Trialists’ Collaborative Group 2000; Gasparini et al. 1991; Rose et al. 1989). An open question in the radiotherapy planning for early breast cancer is the potential toxicity on the heart even if cardiac damage is correlated to the heart-absorbed dose and differs between left- and right-breast radiation therapy (Gagliardi et al. 1996). Some factors, as age, hypertension, diabetes mellitus, total cholesterol, family history of early myocardial infarction at age <60 years, smoking, may influence the individual patient’s risk of developing heart disease (Wilson et al. 1998; Grundy et al. 1999). Systemic therapies used for the treatment of early breast cancer such as anthracyclines, trastuzumab, taxanes, tamoxifen, and letrozole have increased over the last few decades and these may potentiate the radiation’s effects on the heart (Offersen et al. 2011).

The predominance of ischemic heart disease indicates that the coronary arteries and in particular the left anterior descending artery (LAD) may be the critical structure for development of late radiation induced heart morbidity. The anterior portion of the heart and the LAD territory are the cardiac area more often enclosed within the tangential radiation fields used to treat left breast cancer (Schultz-Hector and Trott 2007). Different strategies to minimize dose to the heart are being developed, such as the use of respiratory gated radiotherapy, or alternative positioning of the patient in prone position (Vikstrom et al. 2011; Kirby et al. 2011; Kirby et al. 2010). Although new techniques, including intensity-modulated radiotherapy (Hurkmans et al. 2002) combined with free breathing gating (Korreman et al. 2006) and helical tomotherapy (Hui et al. 2004), may further reduce radiation-induced cardiac toxicities (Prosnitz et al. 2005), the most important factors in limiting cardiac radiation are associated with the techniques used and the expertise of the radiation oncologist. The dose distribution in the heart is not homogeneous and the highest doses are likely to be delivered to the anterior heart, including the LAD. The Danish Breast Cancer Cooperative Group (DBCG)introduced a national guideline recommending the doses to the heart has tobe as low as possible and preferably result in a maximum of 20 Gy can be given to the LAD (Offersen et al. 2010). The aim of the work was to identify, from the study of the different thoracic conformation of patients, a numeric value predictive of increased dose on anterior descending branch of LAD.

## Methods

We have selected ninety consecutive Caucasian females (median age 57.9), treated with radiotherapy for early left breast cancer, pathologic stage I–II, from January to December 2012 (Table 1).

**Table 1:** Characteristics of patients

Characteristics	Parameters	Values
Age	Mean	57.9
	Median	58
	Range	(36–88)
Breast side	Left	91 (100 %)
Pathological tumor stage	Tis	4 (5 %)
	T1	64 (70 %)
	T2	22 (24 %)
	T3	1 (1 %)
Pathological node stage	N0	60 (66 %)
	N1	31 (34 %)
Stadio	0	4 (5 %)
	I	55 (60 %)
	II	32 (35 %)
Surgery	Conservative surgery + biopsy of sentinel node	60 (66 %)
	Conservative surgery + axillary node dissection	31 (34 %)
Histology	Ductal Infiltring Carcinoma	74 (81 %)
	Lobular Infiltring Carcinoma	8 (9 %)
	Mixed Carcinoma	5 (6 %)
	In-Situ Ductal Carcinoma	4 (4 %)

All patients underwent conservative surgery and adjuvant radiotherapy on whole breast (with or without boost on tumor bed) and without indication for regional nodal irradiation ≤3 positive nodes).

For each patient a CT simulation (Siemens somatom) was realized in supine position. Target Volumes were delineated according to the International Commission of Radiation Units criteria (International Commission of Radiation Units and Measurements - ICRU 1993; International Commission of Radiation Units and Measurements - ICRU 1999).

The target breast volume (clinical target volume; CTV) was extended from the clinical image plus second rib insertion in cranial direction, to the loss of CT apparent breast in caudal direction and skin in anterior direction. Pectoral muscles, chest-wall muscles and sternal-rib junction were excluded. Lateral and medial border were highly variable depending on breast size and the amount of ptosis in the supine position. The planning target volume (PTV) was generated from the CTV volume by adding a 7 mm margin in all directions and excluding the skin.

The LAD was contoured separately from the heart, using mediastinal CT windows. The LAD is difficult to visualize in a CT scan because its location is inferred from the course of the anterior-interventricular groove, conseguentely we added a 10-mm axial margin to the LAD to allow for delineation uncertainty and respiratory/cardiac motion (i.e. LAD + 1 cm).

Whole breast irradiation 3D-conformal treatments provided two equally-weighted opposed tangential fields using beams of photons 6 mV. The dose distribution was calculated and optimized using Pinnacle^®^ System.

**Fig. 1:**
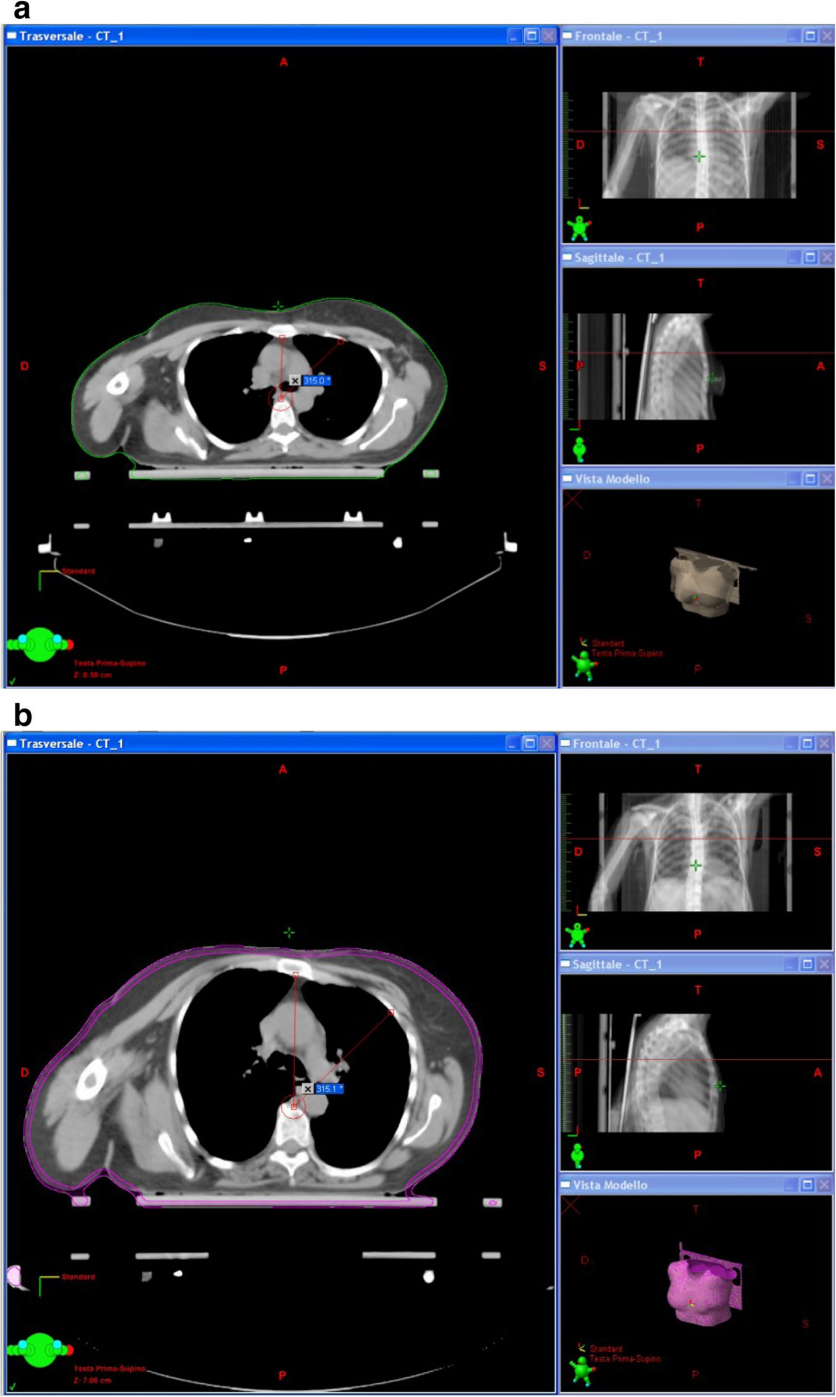
Examples of measurement of the distance **a** and **b**

**Fig. 2:**
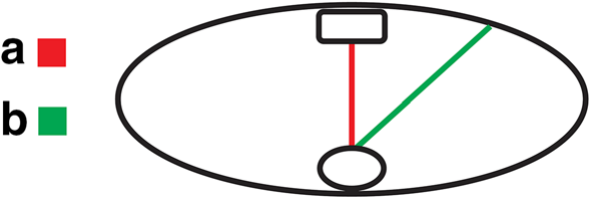
Graphical representation of *a* and *b*

All patients were treated with a standard fractionation of dose: 50 Gy (2 Gy/fraction) to whole breast, and, for stage T1–T2–T3–N0–N1, a sequential additional dose of 10 Gy (2 Gy/fraction) directed to original tumor site. For each patient we calculated the Dose-Volume Histograms (DVHs) to the target and organs at risk focusing on heart and LAD. For each plan on the CT-images we measured the distance between the posterior surface of the sternum and the anterior surface of body of the corresponding vertebra (a), and the distance measured on the line at 45°, between the anterior surface of vertebral body and the back face of the corresponding rib (b) Figs. 1a–b and 2.

We calculated the median values of a/b measured in all patients and we used the value obtained as the cut-off to divide the patients into two groups. In the two groups, we calculated: cardiac Volume (cc); LAD volume with expansion of 0.6 mm to consider moving the LAD resulting from the heart beat and breathing movements PRV LAD (cc); Dmean LAD (Gy); Dmax LAD (Gy); V10 (%) LAD; V20 (%) LAD; V30 (%) LAD. We correlated these values with parametric and non-parametric test (Pearson–Kendall and Spearman test).

## Results

**Fig. 3:**
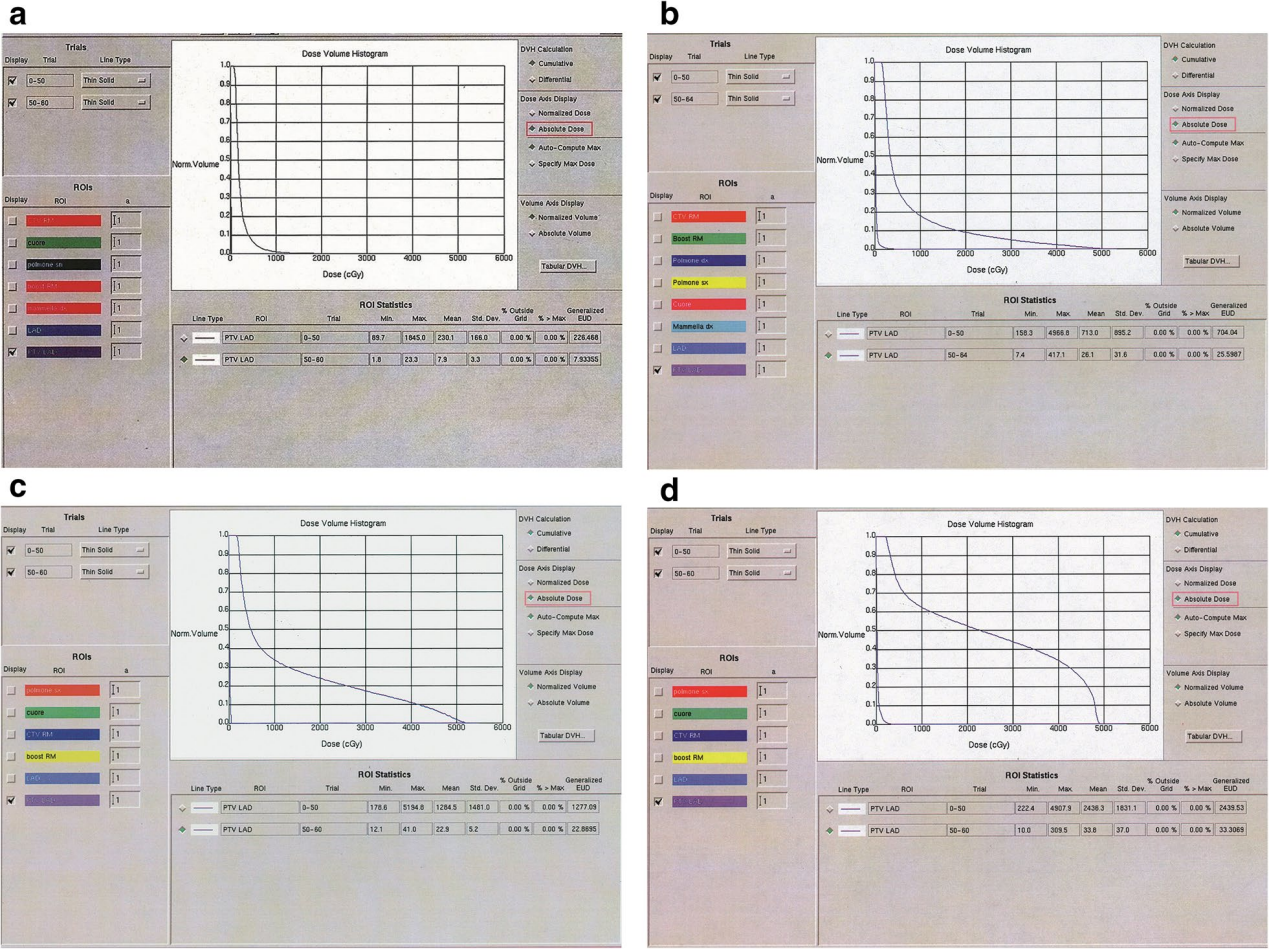
**a** DVH of patient with a/b: 0.747 and V10 PRV(LAD):1.28 %; V20–30–40–50 PRV(LAD): 0; **b** DVH of patient with a/b: 0.634 and V10 PRV(LAD):18 %; V20 PRV(LAD):9.3 %; V30 PRV(LAD): 4.8 %; V40 PRV(LAD): 2.9 %; V50 PRV(LAD): 0; **c** DVH of patient with a/b: 0.756 and V10 PRV(LAD):33.2 %; V20 PRV(LAD):24.8 %; V30 PRV(LAD): 17.7 %; V40 PRV(LAD): 11.6 %; V50 PRV(LAD): 2.1; **d** DVH of patient with a/b: 0.954 and V10 PRV(LAD):53.2 %; V20 PRV(LAD):47.2 %; V30 PRV(LAD): 42.9 %; V40 PRV(LAD): 36.4 %; V50 PRV(LAD): 8 %

The a/b ratio showed values between 0.626 and 1.123. The median value was 0.821 and was used to divide the patients in two groups. Group I included 46 patients with a/b > 0.821 (0.822–1.123). Group II included 45 patients with a/b ≤ 0.821 (0.626–0.821). The first correlation that we have performed was between breast volume and V10–V20–V30 (LAD) in order to evaluate whether the breast volume could affect the dose to the LAD with the angle of the beams needed to cover the target (Fig. 3a–d)

The data concerning the values of Vol breast and values of the ratio a/b are reported respectively in Table 2.

**Table 2:** Volume breast—ratio a/b

Breast volume		a/b	
*N°*	91	*N°*	91
Media	709.67 cc	Media	0.82533
Median	660.23 cc	Median	0.82100
SD	342.51	SD	0.085489
Range	137.6–2095.7 cc	Range	0.626–1.123

We found with the Pearson test a statistically significant correlation between Vol breast and V10, V20 and V30 with borderline significance (p = 0.006; p = 0.02; p = 0.05, respectively). The data were confirmed by non-parametric test, such as Kendall (tau = 0.004; tau = 0.015; tau = 0.016) and Spearman (rho = 0.003; rho = 0.016; rho = 0.015). We conducted categorizing into quartiles of breast volume evaluated the correlation with a/b (Table 3).

**Table 3:** Volume breast categorized into quartiles

	a/b		Total
	≤0.0821	>0.0821	
Vol breast IQ	17 (37 %)	6 (13.3 %)	23 (25.3 %)
IIQ	8 (17.4 %)	15 (33.3 %)	23 (25.3 %)
IIIQ	14 (30.4 %)	9 (20 %)	23 (25.3 %)
IVQ	7 (15.2 %)	15 (33.3 %)	22 (24.2 %)
Total (%)	46 (100 %)	45 (100 %)	91 (100 %)

We found significance (p = 0.01) between small Vol breast and a/b ≤ 0.0821; a greater Vol breast has a greater correlation with a/b > 0.0821.

Finally, we evaluated the distribution V10 in the two groups taking account of the Dmean ≤5 or >5 and we found significance with a/b. Chi square 0.009 (0.01) (Table 4). Therefore patients with a/b ≤ 0.0821 have significant correlation with Dmean ≤5. Patients with a/b > 0.0821 have significant correlation with Dmean >5.

**Table 4:** Chi square

	Value	df	Sig. asint. (2)	Sig. correct (2)	Sig. correct (1)
Chi square of Pearson	6.904	1	0.009		
Continuity correction	5.838	1	0.016		
Likelihood ratio	6.996	1	0.008		
Fisher test				*0.011*	0.008
Association linear–linear	6.828	1	*0.009*		
Number of valid cases	91				

## Discussion

Cardiac disease has been observed in patients receiving radiation to left chest wall after a left-side mastectomy, or due to irradiation on internal mammary chain. In other hand, modern radiotherapy techniques deliver less radiation to the heart than those of 30 years ago even to patients with left-side tumors (Jemal et al. 2011; Violet and Harmer 2004). The predominance of ischaemic heart disease in the anterior area indicates that the coronary arteries and in particular the LAD may be the critical structure for development of late radiation induced heart morbidity. In radiotherapy of breast cancer, the range of doses to the heart has changed due to the development of new techniques, beam energy, target doses, different volumes, and contouring modalities, nevertheless many studies report that the mean cardiac dose is greater for left- thus right-breast irradiation (Taylor et al. 2009; Roychoudhuri et al. 2007; Harris et al. 2006). However, our knowledge regarding the risk factors modulating the acute affects of cardiac radiation are not still sufficient, data regard are required (Darby et al. 2010). It appears that the cumulative dose and its fractioning determine acute and chronic cardiac effects of radiation therapy. In the past, pericarditis used to be the most common side effect in patients receiving traditional radiotherapy for Hodgkin’s disease (Carver et al. 2007). Dose restriction to 30 Gy with lower daily fraction, different weighting of radiation fields, and blocking of the subcarinal region have been reported to reduce the incidence of pericarditis from 20 to 2.5 %. The radiation may cause two types of cardiovascular disease: micro-vascular and macro-vascular disease. The first one is characterized by a decrease in capillary density causing chronic ischaemic heart disease and focal myocardial degeneration, and second one by the faster development of age-related atherosclerosis in the coronary arteries (Schultz-Hector and Trott 2007). However, there are patients with unfavourable anatomy combined with a tumor bed near to the heart who receive a high dose to the heart. Furthermore the aggressiveness of the adjuvant therapy of early breast cancer has increased over the last decades, thus the individual patient is often treated with several systemic therapies which may potentiate the radiation effects to the heart.

Our study, conducted on 91 patients selected for early left breast cancer, has identified a numeric value predictive of increased dose on anterior descending branch of LAD, from the different thoracic conformation of patients. The results have demonstrated that the geometric data a/b and its value >0.0821 could be factors to be taken into account in the enrollment of left breast cancer patients, especially for use of new fractionation schemes in the treatment of breast cancer stage I–II and could be indicative towards a prone set-up or a different technique for these patients with a series of controls cardiac close together. The analysis of the Early Breast Cancer Trialists’ Collaborative Group (Paszat et al. 1998; Darby et al. 2005) on the cause specific mortality among 20,000 women at 10–20 years after primary treatment clearly demonstrated the effectiveness of adjuvant radiotherapy for the reduction of local recurrence by about a factor of 3. However, this benefit did not translate into any survival benefit because it was offset by a statistically significant increase (about 30 %) in the annual death rate from cardiovascular deaths, which was ascribed to inadvertent irradiation of the coronary arteries, the carotid arteries, and other major arteries. Then there is convincing evidence that radiation doses lower than 10 % of the doses usually noted as tolerance doses for the irradiated heart in radiotherapy are associated with a significant increase in cardiovascular morbidity after latencies of 10 years.

The problem of clinical research into radiation-induced cardiovascular risk is the extremely long latency to symptomatic disease. The early perfusion changes which can be precisely quantified and recorded anatomically with modern non-invasive imaging techniques may prove ideal for the way forward. These changes occur early enough to develop, for example criteria of treatment plan optimization in the radiotherapy of breast cancer patients. A comprehensive clinical and translational research program should address a range of open questions, all of which may have an impact on the choice and delivery of post-operative radiotherapy techniques: left breast irradiation in patients with early breast cancer involves radiation-induced cardiovascular disease risk. Although modern radiotherapy techniques are likely to reduce the prevalence and severity of heart disease, her incidence is expected to increase in cancer survivors who have received old radiotherapy regimens. The adequate strategy for screening of heart disease remains a source of debate in the radiation and medical oncology community (Lancellotti et al. 2013). Nieder et al. (2007), claim that IMRT techniques are able to reduce radiation dose to heart and coronary arteries. The identification of patients that for thoracic conformation have greater risk of higher dosage to cardiac structures could be an important factor for discriminating type of treatment, of fractionation and type of positioning in radiation therapy. Pili G. et al. had identified a method to evaluate probability of late cardiac mortality, in the maximal heart distance (the maximal distance from the posterior edge of tangent field to the heart contour). However, no clear association was found between the maximal heart distance and cardiovascular disease risk, but assert that it is important to modify the treatment technique to decrease the irradiate cardiac volumes and reduce the potential risk of late RT-associated cardiac complications. The authors conclude that the preventive assay of geometric parameterization of the thorax could permit clinicians to choose and propose the safest therapeutic strategy for the patients (Pili et al. 2011; Cammarota et al. 2014). As in our work, we looked for a correlation between the distance of the posterior surface of the sternum and the anterior surface of body of the corresponding vertebra, and the distance measured on the line at 45°, between the anterior surface of vertebral body and the back face of the corresponding rib. We found, with a statistically analysis, a significant correlation between Vol breast and V10, V20 and V30 with borderline significance (p = 0.006; p = 0.02; p = 0.05, respectively). We found, also, the significance (p = 0.01) between small Vol breast and a/b ≤ 0.0821; a greater Vol breast has a greater correlation with a/b > 0.0821.

The anatomical variability is reflected by the volume range of the organs at risk included in the treatment of breast. The prone position enabled lower mean doses to the heart and lung, as compared with the supine position. Although techniques such as partial breast irradiation seems to be the safest and least likely to have cardiovascular events in the future. It’ important the accurate selection of the patient, based on the disease stage but also on the anatomy, intended not only on the breast volume, but also on the conformation of the chest.

We think our work can contribute to find new elements able to offer each patient the most appropriate customized treatment.
